# Two years follow-up of golimumab treatment in refractory enteropathic spondyloarthritis patients with Crohn disease: A STROBE-compliant study

**DOI:** 10.1097/MD.0000000000025122

**Published:** 2021-03-26

**Authors:** Paola Conigliaro, Maria Sole Chimenti, Paola Triggianese, Arianna D’Antonio, Giorgia Sena, Norma Alfieri, Livia Biancone, Roberto Perricone

**Affiliations:** a Rheumatology, Allergology and Clinical Immunology; b Gi Unit, Department of “Medicina dei Sistemi”, University of Rome Tor Vergata, Rome, Italy.

**Keywords:** Crohn disease, enteropathic spondyloarthritis, golimumab, tumor necrosis factor

## Abstract

Golimumab is a fully human monoclonal antibody against tumor necrosis factor (TNF) approved for the treatment of ulcerative colitis and not for Crohn disease (CD). Many CD patients experience primary, secondary failure, or intolerance to other TNF inhibitors (TNFi) approved in Italy for CD (adalimumab and infliximab). Spondyloarthritis (SpA) may be associated with CD (enteropathic, ESpA) in up to 50% of patients requiring a multidisciplinary and tailored approach. However, only few data from literature and no formal trials determined the efficacy and safety of golimumab in ESpA patients. We performed a case series on 12 patients affected by active CD and active ESpA were failure or intolerant to previous TNFi approved in Italy for both SpA and CD, infliximab and adalimumab. Golimumab was administered following rheumatologic dosage (subcutaneous 50 mg monthly; 100 mg monthly for patients ≥100 kg). Gastrointestinal and rheumatologic disease activity was evaluated with a follow-up of 2 years. A total of 9 patients were followed for 2 years of golimumab treatment. CD clinical activity ameliorated as shown by the reduction of Harvey–Bradshaw index and Crohn disease activity index (CDAI) at 12 and 24 months of treatment (*P* = .03 and *P* = .04, respectively) associated with reduction of C-reactive protein at 12 and 24 months (*P* = .04 for both comparisons) of treatment. SpA assessment revealed a significant reduction in tender joint count at 6 (*P* = .03), 12 (*P* = .03), and 24 months (*P* = .007) of treatment. Swollen joint count, pain, SpA disease activity, and disability reduced in several patients during the follow-up. No adverse events were registered in the follow-up. We demonstrate good clinical efficacy and safety profile of both gastrointestinal and rheumatologic involvement. This may indicate promising therapeutic option for ESpA patients affected by CD, and non-responsive to other TNFi.

## 1 Introduction

Inflammatory bowel disease (IBD) includes Crohn disease (CD) and ulcerative colitis (UC), characterized by chronic intestinal inflammation frequently associated with extra-intestinal manifestations such as arthritis.^[1]^ The association of IBD and Spondyloarthritis (SpA, enteropathic SpA—ESpA) has a prevalence up to 50% and requires an integrated approach for the management.^[2]^ Treatment strategy may require a patient tailored-approach since primary non-response, secondary loss of response, or intolerance occur. Among the “big-five” family of tumor necrosis factor (TNF)-inhibitors (TNFi), adalimumab and infliximab are approved in Italy for CD. Certolizumab received approval by Food and Drug Administration while it was refused by EMA limiting its employment in Europe (https://www.ema.europa.eu/en/medicines/human/EPAR/cimzia#application-details-section). Golimumab, fully human IgG1k monoclonal antibody against TNF, is approved for the treatment of IBD only for moderate-to-severe active UC and not for CD. The efficacy of golimumab in patients with CD has been investigated only in few studies and no formal trials assessed its efficacy. In particular, 2 small case series and 3 retrospective studies explored the use of golimumab in CD patients, however only few patients with ESpA were included.^[3–8]^

Aim of this study was to investigate both gastroenterological and rheumatologic clinical efficacy and safety profile of golimumab in a small cohort of Italian CD and ESpA patients refractory to previous TNFi infliximab and adalimumab. A review of the literature of previous cases was performed.

## 2 Case series

Observational cohort study on 12 patients with moderate to severe ESpA and refractory CD receiving golimumab treatment. Consecutive patients were prospectively enrolled in the combined GI–Rhe clinic of the University of Rome Tor Vergata from January 2018 to January 2019. Eligibility criteria were: diagnosis of CD and SpA established using standard European Crohn's and Colitis Organisation and Assessment of SpondyloArthritis international Society criteria; failure to previous TNFi adalimumab and infliximab; available data at follow-up. Golimumab was administered following rheumatologic dosage (subcutaneous 50 mg monthly; 100 mg monthly for patients ≥100 kg). Clinimetric indexes and inflammatory markers for joint and gastrointestinal involvement were evaluated at baseline and after 6, 12, and 24 months of treatment. Primary outcome was clinical gastrointestinal and rheumatologic response after starting golimumab. Gastrointestinal response was evaluated by Harvey–Bradshaw index (HBI) and Crohn disease activity index (CDAI) while rheumatologic response was assessed with tender and swollen joint count. Secondary outcomes were modification of laboratory markers C-reactive protein (CRP) and erythrocyte sedimentation rate (ESR); SpA disease activity measured by composite index ankylosing spondylitis disease activity score (ASDAS) and bath ankylosing spondylitis disease activity index (BASDAI); patient reported outcomes pain-visual analogue scale (VAS) and patient global assessment (Pt-GA); disability evaluated by health assessment questionnaire (HAQ); safety of golimumab. This study was approved by the local Ethics Committee. Written informed consent was obtained from the patients. Data in the longitudinal analysis during the treatment course of individual patients were evaluated with the non-parametric Wilcoxon signed-rank test. *P*-values <.05 were considered significant. Statistical analyses were performed using GraphPad Prism version 6 (GraphPad Software, Inc., San Diego, CA).

**Figure 1:**
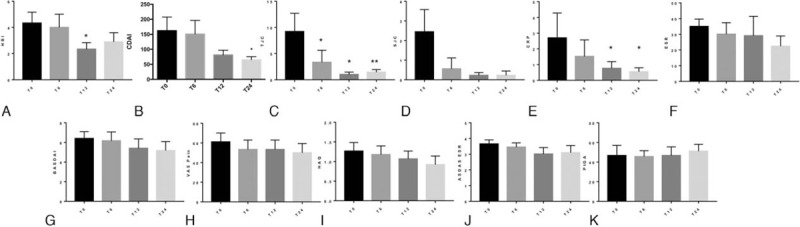
Clinical and laboratory assessment in the study population. (A–B) Modification of Harvey–Bradshaw index (HBI) and Crohn disease activity index (CDAI) in patients with Crohn disease and enteropathic spondylarthritis (SpA) in the follow-up. (C–D) Tender joint count (TJC) and swollen joint count (SJC) at baseline (T0), 6 (T6), 12 (T12), and 24 (T24) months of golimumab treatment. (E) Significant reduction of C-reactive protein (CRP) levels at T12 and T24 of follow-up. (F) Erythrocyte sedimentation rate (ESR) in the follow-up. (G) Clinimetric index of SpA disease activity bath ankylosing spondylitis disease activity index (BASDAI). (H) Assessment of pain by visual-analogue scale (VAS), and (I) disability by health assessment questionnaire (HAQ). (J) SpA disease activity measured by ankylosing spondylitis disease activity score (ASDAS), and (K) patient global assessment (PtGA). ^∗^*P* < .05, ^∗∗^*P* < .01.

A total of 9 patients achieved 24-months of follow-up. Three patients were excluded since data were not available at all time points. All patients were refractory or intolerant to 2 adalimumab and infliximab for both SpA and CD. Demographic, clinical characteristics, and previous medications of all patients are summarized in Table 1. CD clinical activity, assessed by HBI, significantly reduced at 12 months (from 4.33 ± 2.35 at T0 to 2.33 ± 1.5 at T12; *P* = .03) of treatment (Fig. 1A). CDAI were also reduced at 24 months (*P* = .04, Fig. 1B). Rheumatologic assessment showed significant reduction of tender joint count after 6 (*P* = .03), 12 (*P* = .03), and 24 months (*P* = .007) of treatment (Fig. 1C). Swollen joint count reduced over time without reaching a statistically significance (Fig. 1D). No cases of dactylitis were observed at baseline and during the follow-up. A reduction of CRP (mg/dL) after 12 and 24 months (2.7 ± 4.49 at T0, 0.75 ± 1.2 at T12, and 0.54 ± 0.71 at T24; *P* = .04 for both comparisons) of treatment was observed while ESR remained stable (Fig. 1E and F). SpA disease activity measured by BASDAI and patients reported outcomes pain-VAS and disability by HAQ slightly reduced during the follow-up (Fig. 1G–I). ASDAS and Pt-GA remained stable in the follow-up (Fig. 1J and K). No adverse events were observed during the 24 months follow up.

**Table 1:** Clinical characteristics of patients enrolled.

Case	1	2	3	4	5	6	7	8	9
Age, y	50	49	50	23	73	41	65	46	57
Sex	F	F	F	F	F	F	M	M	M
Age at diagnosis, y	41	49	42	20	36	37	49	19	23
Smokers	Former smoker	Former smoker	No	No	Former smoker	No	Former smoker	Former smoker	Yes
Montreal classification	A3, L1, B1	A3, L3, B1 p	A3, L2, B1 p	A1, L2, B1	A2, L1, B2	A2, L1, B1	A3, L1, B1	A2, L1, B2	A2, L1, B3
HLA B27	Negative	Negative	Negative	Negative	Positive	Negative	Negative	Positive	Positive
Fistulae	No	No	No	No	No	No	No	No	No
Stenosis	No	No	No	No	Yes	No	No	Yes	No
ESpA	Peripheral SpA	n-rx axial and peripheral SpA	Peripheral SpA	Peripheral SpA	AS	AS	AS	AS	AS
Extraintestinal manifestations	EN	PsO	PsO	EN	No	PsO, EN, anterior uveitis	Anterior uveitis	No	No
Previous medication									
Steroids	+	+	+	+	+	+			
Azathioprine		+	+		+				+
6-Mercaptopurin			+					+	
Mesalazine								+	
Methotrexate	+		+		+	+			
Sulphasalazine	+		+	+		+	+		+
Infliximab	+	+	+	+	+	+	+	+	+
Adalimumab	+	+	+	+	+	+	+	+	+
Surgery Prior to golimumab	Ileostomy	Proctocolectomy and ileostomy	Perianal fistulae resection	−	Ileostomy	−	−	−	Ileocecal resections/ proximal ileum stricture pasty
During golimumab treatment	−	Perianal abscess and fistulae	−	−	−	−	−	−	−
Cause of failure to previous TNFi Infliximab	Adverse event (Urticaria)	Primary failure on CD	Adverse event (upper airway oedema)	Primary failure on CD and ESpA	Adverse event (fever, dyspnoea)		Adverse event (recurrent infections)	Secondary failure on CD	
Adalimumab	Secondary failure on ESpA	Primary failure on CD	Adverse event (urticaria)	Secondary failure on ESpA	Secondary failure on ESpA	Adverse event (upper airway oedema and urticaria)	Secondary failure on ESpA	Adverse event (urticaria)	

A1, below 16 years; A2, between 17 and 40 years; A3, above 40 years; L1, ileal; L2, colonic; L3, ileocolonic; B1, non-stricturing; B2: structuring; B3, penetrating, P, perianal disease. AS = ankylosing spondylitis, CD = Crohn disease, EN = erythema nodosum, PsO = psoriasis, SpA = spondyloarthritis.

## 3 Discussion

We demonstrate good clinical efficacy and safety profile of both gastrointestinal and rheumatologic involvement. This may indicate promising therapeutic option for ESpA patients affected by CD and non-responsive to other TNFi. Although TNFi have the same target, switching from a different molecule may be effective.^[2]^ However, this issue has been explored only in few cases of ESpA patients with CD and in none of them from both the gastroenterological and rheumatologic point of view (Table 2). Herein, in our case-series, ESpA patients were failure to 2 TNFi for both joint and intestinal manifestations. We observed persistent improvement in both diseases, as indicated by clinimetric indexes and inflammatory parameters. Our findings are supported by a French retrospective large study including 115 CD patients of which 38 displayed a concomitant SpA assessed in a median follow-up of 9.8 months.^[4]^ In this study clinical response was observed in 56% of CD patients while discontinuation was detected in 6% of patients because of intolerance.^[4]^ Recently, a Swedish registry explored the efficacy of golimumab in 94 CD patients demonstrating a retention rate of 85% at 12 weeks, however concomitant SpA was not reported.^[6]^ Though Fiehn and Vay^[5]^ described a paradoxical effect of golimumab: CD patients with associated SpA experienced IBD flares after golimumab. Mechanism of this effect remains unclear, although an imbalance of cytokines with increased production of interferon-α in genetically predisposed individuals has been suggested. In a small case series of 8 patients with refractory CD, authors retrospectively demonstrated a potential clinical effect in selected subgroup of difficult-to-treat CD patients.^[2]^ In particular, 4 patients had a concomitant SpA and golimumab was effective in 2 of them while residual clinical symptoms and paradoxic psoriasis were observed in the other 2 patients.^[2]^

**Table 2:** Treatment with golimumab in Crohn disease patients.

Reference	N of CD patients	N of CD patients with SpA	Median duration treatment (IQR)	Primary outcome	Secondary outcomes
^[4]^	115	38	9.8 months (0.5–44)	Median duration of GLM: 9.8 months	Decrease HBI of 3 points: 55.8%
^[3]^	8	4	nd	Clinical response 37.5%	Adverse events: 1 case psoriasiform pustulosis
^[6]^	94	0	89 weeks (32–158)	Drug retention rate at 12 months: 85.1%	Predictors of discontinuation at 12 weeks: surgery, corticosteroid, female sex
^[7]^	6^∗^	0	9 months (5–18)	Clinical response: 50% suboptimal response	Discontinuation: 3 patients
^[8]^	45	0	22 months (12–34)	Clinical response at 3 months: 77.7%	Rates of sustained clinical response at 12 and 36 months: 81% and 64%; endoscopic response: improvement 73% and mucosal healing 43%, predictive factors of response at 6 months: high CRP, high maintenance dose; adverse events: 33%

CD = Crohn disease, CRP = C-reactive protein, SpA = Spondyloarthritis.

∗ Pediatric patiens.

Our study encompasses several limitations including small group of patients with potential bias of selection and not allowing a proper assessment of the rate of response and remission, single-center design, and lack of IBD endoscopic evaluation.

In conclusion, golimumab effect at rheumatologic dosage is suggested in a peculiar group of active ESpA and active CD patients showing failure to other TNFi. Long-term follow-up of 24 months support our hypothesis that golimumab may be a good opportunity in these patients although data in randomized controlled trials and large real-world evidence are needed to confirm our findings.

## Author contributions

**Conceptualization:** Paola Conigliaro, Maria Sole Chimenti, Livia Biancone, Roberto Perricone.

**Data curation:** Paola Triggianese, Arianna D’Antonio, Giorgia Sena, Norma Alfieri.

**Formal analysis:** Paola Conigliaro, Maria Sole Chimenti, Paola Triggianese, Arianna D’Antonio.

**Investigation:** Paola Triggianese, Arianna D’Antonio, Giorgia Sena, Norma Alfieri.

**Methodology:** Paola Conigliaro, Maria Sole Chimenti, Paola Triggianese, Arianna D’Antonio, Giorgia Sena, Norma Alfieri.

**Project administration:** Paola Conigliaro, Maria Sole Chimenti, Paola Triggianese, Livia Biancone, Roberto Perricone.

**Supervision:** Paola Conigliaro, Maria Sole Chimenti, Livia Biancone, Roberto Perricone.

**Writing – original draft:** Paola Conigliaro, Maria Sole Chimenti, Arianna D’Antonio.

**Writing – review & editing:** Paola Conigliaro, Maria Sole Chimenti, Livia Biancone, Roberto Perricone.
